# Divergent adherence estimates with pharmacokinetic and behavioural measures in the MTN-003 (VOICE) study

**DOI:** 10.7448/IAS.19.1.20642

**Published:** 2016-02-04

**Authors:** Ariane van der Straten, Elizabeth R Brown, Jeanne M Marrazzo, Michael Z Chirenje, Karen Liu, Kailazarid Gomez, Mark A Marzinke, Jeanna M Piper, Craig W Hendrix, on behalf of the MTN-003 VOICE Protocol Team for the Microbicide Trials Network

**Affiliations:** 1 Women's Global Health Imperative (WGHI) RTI International, San Francisco, CA, USA; 2 Center for AIDS Prevention Studies (CAPS), Department of Medicine, University of California San Francisco, San Francisco, CA, USA; 3 Fred Hutchinson Cancer Research Center, Seattle, WA, USA; 4 Departments of medicine and of biostatistics, University of Washington Seattle, WA, USA; 5 University of Zimbabwe-UCSF Research Collaboration, Harare, Zimbabwe; 6 FHI360, Durham, NC, USA; 7 Department of medicine, Johns Hopkins University, Baltimore, MD, USA; 8 NIAID/DAIDS Bethesda, MD, USA

**Keywords:** microbicide, pre-exposure prophylaxis, adherence measurement, pharmacokinetic drug detection, HIV

## Abstract

**Introduction:**

In the Microbicide Trial Network MTN-003 (VOICE) study, a Phase IIB pre-exposure prophylaxis trial of daily oral or vaginal tenofovir (TFV), product adherence was poor based on pharmacokinetic (PK) drug detection in a random subsample. Here, we sought to compare behavioural and PK measures of adherence and examined correlates of adherence misreporting.

**Methods:**

We included participants with PK and behavioural data from VOICE random subsample. Behavioural assessments included face-to-face interviews (FTFI), audio computer-assisted self-interviewing (ACASI) and pharmacy-returned product counts (PC). TFV concentrations <0.31 ng/mL in plasma (oral group) and <8.5 ng/swab in vaginal group were defined as “PK non-adherent.” Logistic regression models were fit to calculate the combined predictive ability of the behavioural measures as summarized by area under the curve (AUC). Baseline characteristics associated with over-reporting daily product use relative to PK measures was assessed using a Generalized Linear Mixed Model.

**Results:**

In this random adherence cohort of VOICE participants assigned to active products, (*N*=472), PK non-adherence was 69% in the oral group (*N*=314) and 65% in the vaginal group (*N*=158). Behaviourally, ≤10% of the cohort reported low/none use with any behavioural measure and accuracy was low (≤43%). None of the regression models had an AUC >0.65 for any single or combined behavioural measures. Significant (*p*<0.05) correlates of over-reporting included being very worried about getting HIV and being unmarried for the oral group; whereas for the vaginal group, being somewhat worried about HIV was associated with lower risk of over-reporting.

**Conclusions:**

PK measures indicated similarly low adherence for the oral and vaginal groups. No behavioural measure accurately predicted PK non-adherence. Accurate real-time measures to monitor product adherence are urgently needed.

Trial registration: ClinicalTrials.gov identifier: NCT00705679

## Introduction

Daily oral pre-exposure prophylaxis (PrEP) is an effective prevention strategy for persons at risk of HIV-1 acquisition, and Truvada^TM^ (tenofovir (TFV) disoproxil fumarate/emtricitabine) has received regulatory approval for PrEP in the United States [1]. Risk of HIV acquisition is reduced by >90% when adherence is high based on plasma drug concentrations [2–4]. Reciprocally, suboptimal adherence explains divergent effectiveness results across PrEP studies, locations and populations [3–11]. Indeed, daily oral PrEP was not found effective in preventing HIV-1 acquisition among high-risk African women enrolled in the FEM-PrEP or VOICE trials where plasma TFV detection was less than 40 and 30%, respectively [6,11].

Daily TFV gel use was not found effective in preventing HIV-1 acquisition in VOICE either, attributed to poor adherence based on levels of detectable TFV in plasma (25%) and vaginal swabs (49%) [11]. Pericoital dosing of TFV 1% gel conferred 39% protection in the CAPRISA 004 trial [12]. However, the FACTS-001 confirmatory trial, where product use was low, was unable to demonstrate TFV gel effectiveness [13]. In secondary analyses in VOICE and FACTS-001, TFV gel conferred modest but significant protection when plasma TFV concentrations indicated use [11,13]. Consistently across HIV PrEP studies, adherence assessed by plasma TFV concentrations, adjusted for oral or vaginal route of dosing, correlated well with HIV protection [14].

Accurately measuring adherence to study products has been an on-going challenge in HIV prevention trials. Self-reports typically overestimate adherence because of various biases, including recall and social desirability, yet alternative measures to assess product use are limited [8,15–17]. Hence, antiretroviral (ARV) drug concentrations have been used in recent PrEP trials as a surrogate for adherence, with appropriate adjustments made for route of administration and HIV exposure route. For example, in an oral TFV-containing regimen, high daily adherence is indicated by plasma concentrations ≥40 ng/mL [14,18,19]. Accordingly, pharmacologic measures of product use in trials have clearly shown a dose–response relationship between drug concentration in a variety of anatomic locations (e.g. plasma, peripheral blood mononuclear cells (PBMC), cervicovaginal fluid) and product efficacy [2,14,20–22].

Recent PrEP trials have highlighted a biological–behavioural adherence gap. It was small (14 to 16% overestimation by self-report) in the TDF2 and Partners PrEP trials, where oral products were protective and all monitoring modalities confirmed high adherence [4,23]. However, this gap was >50% in Fem-PrEP and VOICE, which did not demonstrate product effectiveness [6,11], and it varied regionally in iPrEx, where oral Truvada^TM^ was found protective among men who have sex with men and transgender women [5,24].

Here, we examined different behavioural measures of adherence in the VOICE study and compared them to biological pharmacokinetic (PK) assessment of TFV in plasma (for the two oral arms, referred to henceforth as oral group) and vaginal swabs (for the vaginal gel group), to explore their accuracy, alone or in combination, for predicting biological use. We also explored correlates of adherence over-reporting to identify those at greater risk of concealing low product use.

## Methods

VOICE was a Phase IIB double-blinded, randomized, placebo-controlled trial that tested three products (oral TFV disoproxil fumarate (TDF); oral emtricitabine (FTC)/TDF; and TFV 1% vaginal gel) among 5029 women in Uganda, Zimbabwe and South Africa. As described in the primary publication, the oral TDF and FTC/TDF tablets had differing appearances (different sizes and shades of blue) so to maintain blinding each oral arm participant was assigned to take two tablets a day, TDF or TDF placebo and FTC/TDF or FTC/TDF placebo [11]. Intent-to-treat analysis showed these products were safe but not effective in preventing HIV acquisition. Behavioural assessments included monthly face-to-face interviews (FTFI), quarterly audio computer-assisted self-interviewing (ACASI) and monthly counts of returned unused products at the site's pharmacy (product counts [PC]). Post-trial analysis of PK drug concentrations in plasma and vaginal fluid (VF) collected by vaginal swab was conducted in a random subsample of the participants assigned to active products [11].

### Study sample and selection

**Figure 1:**
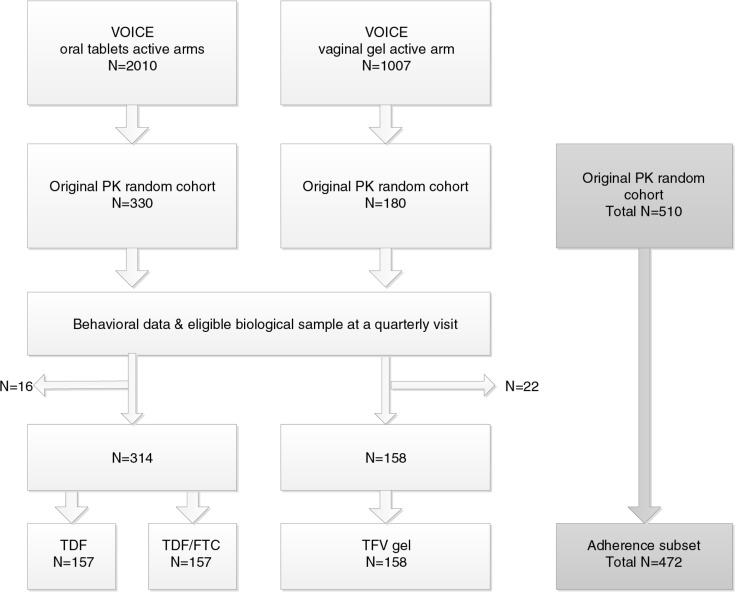
VOICE Adherence Cohort Sample Selection. Sample selection for the adherence cohort among VOICE participants assigned to active arms in the oral and vaginal groups. Sixteen participants in the oral active arms and 22 in the vaginal gel active arm were excluded from the analysis because of missing behavioural data at a visit where biological data were available. PK=pharmacokinetic, TDF=Tenofovir Diproxil Fumarate, FTC=Emtricitabine, TFV=Tenofovir.

Of the participants in the VOICE study who were assigned to active arms (*N*=3017), 510 were included in the original PK random subsample [11], and those who had at least one quarterly visit with complete behavioural and biological data were included in the “adherence cohort” used for this analysis (*N*=472; see Figure 1). Visits were excluded if they occurred after seroconversion or after the data safety and monitoring board (DSMB) recommended oral TDF and gel arms be stopped early (September and November 2011, respectively) because of futility. For the behavioural measures accuracy assessment, we randomly selected one quarterly visit for each participant from our analytical sample from which to draw biological and behavioural adherence assessment data, so that each participant would contribute equally regardless of total number of visits. For the over-reporting assessment, we included all available quarterly visits with biological, FTFI and ACASI data for each participant.

### Measures

#### Biological drug data

“PK non-adherent,” a dichotomous biological outcome, was defined as having a TFV drug concentration below the cut-off value corresponding with no product used in the past week. This adherence threshold is not consistent with protection and was selected only as a surrogate of product adherence behaviours. In the oral group, adherence was assessed quarterly using plasma TFV concentrations (and up to 10 visits were available for plasma sample testing), using a cut-off of 0.31 ng/mL [19,25]. In the gel group, adherence was assessed semiannually using VF TFV concentrations (starting at month six; and up to three visits were available for VF testing), with a cut-off of 8.5 ng/swab based on assessment of TFV VF kinetics derived from several oral and vaginal dosing studies [25–28]. Analysis of TFV in each biological sample has been previously described [11,18].

#### Self-reported adherence

At quarterly visits, frequency of product use in the past seven days, collected both through FTFI and ACASI, was dichotomized into zero versus ≥1 doses in the past week to match the biological measures. During the ACASI, participants were also asked to rate their ability to use the product as instructed in the previous 4 weeks, using a validated six-point rating scale (very low to excellent) [29].

#### Returned PC

Two bottles of 30 pills or a box containing 30 gel applicators were dispensed to participants at baseline and at monthly follow-up visits scheduled at 28-day intervals. At monthly visits, pharmacists collected the number of products returned and dispensed. The difference between the number of products received at the previous visit and the number of products returned represented the number of products assumed used by PC. This number was compared with the number of days that had elapsed between the previous visit and the current visit. The PC was dichotomized as ≤75% use in the past month to most closely match the estimate for PK non-adherence in the past week. In other words, if a participant did not use tablets or gel in the past week, at most she could be 75% adherent by monthly PC.

#### Over-reporting

We defined “obvious” over-reporting when self-report of product used daily in the past seven days by FTFI (or ACASI) occurred with no evidence of use by PK data (plasma for oral group and VF for vaginal group).

### Statistical analysis

We compared characteristics of the adherence subset to the remaining VOICE sample using t-tests for continuous variables and Chi-square tests for categorical variables.

For each behavioural measure, we analyzed its predictive properties for biologically measured PK non-adherence. Accuracy was defined as the proportion of concordant results between the biological and behavioural measures, calculated as the per cent of behavioural measures that were correct given the biological measure. We further explored the ability of the self-reported measures to predict biologically defined adherence by combining the measures to predict PK adherence in a logistic regression model. Using predicted adherence from the logistic regression model as a marker of adherence, we calculated the corresponding area under the ROC curve (AUC) to compare the predictive ability of the single or combined self-reported measures.

We assessed the association between baseline characteristics of participants and obvious over-reporting of product use by self-report (FTFI) versus biological data. For each route of administration, we used a Poisson Generalized Linear Mixed Model (GLMM) with random intercept and included all quarterly visits with complete biological and behavioural data, adjusting for time (in months since enrolment) and country. We estimated the relative risk (RR) ratio for the following group of predetermined baseline characteristics: sociodemographic, sexual behaviour, contraception and HIV risk. We used a validated HIV risk assessment tool (possible values 0 to 13) that predicts HIV acquisition among African women (composed of the following variables: age, marital status, financial support from partner, non-monogamous primary partner, curable STIs, HSV2 status, alcohol use [30]; see Table 1). A cut-off at >6 was chosen as it was associated with annual HIV incidence >10% in VOICE. We *a priori* set the significance level at *p*<0.1 (marginal Wald Chi square test) for entering significant factors into a multivariable model. Sensitivity analyses were conducted assessing characteristics of participants associated with over-reporting at first and second quarter with similar findings (data not presented).

**Table 1:** Baseline characteristics of the adherence cohort (*N*=472) overall and by route of administration, with comparison to the remainder of VOICE participants

	VOICE	VOICE Adherence Cohort			VOICE Participants not in cohort *N*=4557	*p*^5^
		
	Total *N*=5029	Oral *N*=314	Vaginal *N*=158	Total *N*=472		
**Country**						
South Africa	81%	80%	78%	79%	81%	
Uganda	6%	6%	6%	7%	6%	
Zimbabwe	13%	14%	16%	14%	12%	0.46
Mean age (SD)^3^	25.3 (5.2)	25.0 (4.9)	25.5 (5.4)	25.4 (5.2)	25.3 (5.2)	0.65
≥Some secondary school (*)^3^	92%	94%	92%	92%	92%	0.90
Earns own income^3^	42%	45%	49%	47%	42%	0.03
Not married^3^	79%	82%	77%	79%	79%	>0.99
Has main male partner^4^	97%	99%	97%	98%	97%	0.69
Other male sex partners, past 3 months^4^	22%	19%	19%	21%	21%	0.98
No. of sex acts, past 7 days^4^	2.5 (3.1)	2.5 (2.3)	2.3 (2.3)	2.4 (2.4)	2.6 (3.2)	0.20
Condom use, last vaginal sex^4^	85%	85%	81%	85%	85%	0.98
Anal sex, past 3 months^4^	17%	17%	21%	18%	17%	0.86
Sex work in past year^4^	6%	6%	8%	7%	6%	0.68
Contraception method^3^						
Injectable	71%	75%	68%	70%	71%	0.74
Oral contraceptive pills	23%	18%	20%	21%	23%	0.33
Baseline diagnosis of a curable STI^1^	20%	19%	20%	20%	20%	>0.99
HSV-2 seropositivity^2^	46%	44%	39%	43%	46%	0.24
Very/somewhat worried about getting HIV in next year^4^	78%	75%	78%	76%	78%	0.30
HIV risk score above 6 (**)	17%	15%	21%	18%	17%	0.68

* Some secondary education or more.

** Possible values are 0 to 13 and score includes the following baseline predictors of HIV risk: younger age group, not married or living with primary partner, no financial support from partner, primary partner has other sexual partners, curable STI, HSV2-seropositivity and self-reported alcohol use in past 3 months [30].

1 Includes diagnosis for *C. trachomatis, N. gonorrhoeae*, *T. vaginalis* and Syphilis as previously described [11].

2 HSV-2 seropositivity was determined with the HerpeSelect 2 enzyme immunoassay (Focus Technologies) at enrolment; an index value of 3.5 or greater was considered a positive result [11].

3 Collected by face-to-face interviews (FTFI) on case report forms.

4 Collected by audio computer-assisted self-interviewing (ACASI).

5 *P*-values are derived from t-test for age (continuous variable) and Chi-square tests for the categorical variables, aiming to provide evidence for whether participants are differentially included in the adherence subset.

All participants provided written informed consent prior to any study procedures. The VOICE study was approved annually by all institutional review boards and ethics committees at all participating institutions in Zimbabwe, South Africa and Uganda and was overseen by the regulatory infrastructure of the US National Institutes of Health and the Microbicide Trials Network [11].

## Results

### Study sample

This adherence cohort included 472 participants allocated to active arms in VOICE: 157 in the TDF arm, 157 in the TDF/FTC arm and 158 in the TFV gel arm (Figure 1). There were a total of 1350 quarterly visits (median: month-9 visit; range: month-3 to month-30 visit). Participants in the oral group completed a median of three visits (range 1 to 10) and those in the gel group completed a median of one visit (range 1 to 3).

Table 1 displays baseline characteristics of participants in the adherence cohort by route of administration and compared to the full VOICE sample. Overall, participant mean age was 25.4 years; 98% had a main male sex partner; 79% were unmarried; and 79, 14 and 7% were from South Africa, Zimbabwe and Uganda, respectively. The adherence cohort was similar to the full VOICE sample except that those in the cohort were more likely to earn their own income compared with the remainder VOICE sample (*p*=0.03).

### Evidence of non-adherence by route of administration and by different measures

**Figure 2:**
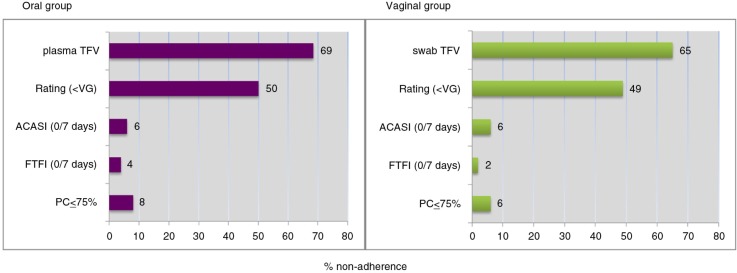
Evidence of non-adherence by route of administration and by different measures. Non-adherence in oral (*N*=314) and vaginal (*N*=158) groups is presented for the following measures: Plasma TFV level (oral group; plasma TFV) or VF TFV level from vaginal swab (vaginal group, swab TVF); audio computer-assisted self-interviewing (ACASI) 6-point self-rating scale assessing ability to use in the past month, dichotomized as less than very good versus very good/excellent (Rating <VG); ACASI reports of 0 doses in the past seven days (ACASI 0/seven days); face-to-face interview (FTFI) reports of 0 doses in the past seven days (FTFI 0/seven days); unused products returned to the clinic corresponding to 75% or less adherence in the past month (PC ≤ 75%).

For the accuracy assessment, we randomly selected one quarterly visit for each participant (median; month-6 visit, range months 3 to 30 visit). Sixty-nine per cent of those in the oral group and 65% of those in the vaginal group exhibited drug levels below the PK non-adherence cut-off. In contrast, by FTFI, 4% of participants in the oral group and 2% in the gel group reported not taking any dose in the past week. By ACASI, this was 6% in both groups. Based on the PC measurement, 8 and 6% of participants in the oral and the vaginal groups were classified as non-adherent, respectively (Figure 2).

**Figure 3:**
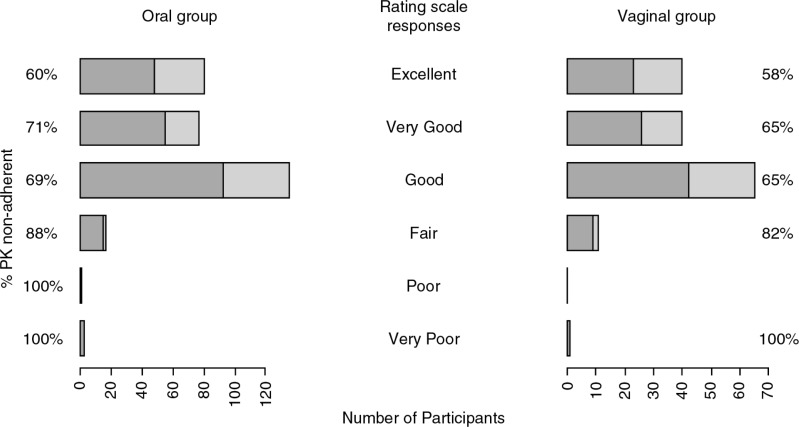
Participants’ responses to the ACASI self-rating scale by PK non-adherence level. During quarterly audio computer-assisted self-interviewing (ACASI), participants completed a validated product adherence self-rating scale that asked, “Please rate your ability, over the past 4 weeks, to [take tablets/insert gel] exactly as you were instructed.” Participants could select one of six response categories (from very poor to excellent). The count of participants for each response category is displayed for the adherence subset (with one random quarterly visit selected for each participant) by route of administration. Within each response category, the percentage who were PK non-adherent (had no evidence of product use in the past seven days) is indicated in dark grey.

When asked by ACASI to rate their adherence in the past 4 weeks from very poor to excellent, the majority of women reported high adherence regardless of biological evidence (Figure 3).

Accuracy by FTFI, ACASI or PC was low: in the oral group, of the 11 participants who reported taking no doses in the past week by FTFI, all were PK non-adherent. However, among the 295 (96%) who reported ≥1 dose in the past week by FTFI, 67% were found to be PK non-adherent (36% accuracy). Accuracy was also low by ACASI (38%) and by PC (38%). Accuracy was similarly low in the gel group for the three behavioural measures (39, 41 and 43%, respectively; see Table 2). Sensitivity analyses were conducted for these accuracy estimates (see Supplementary File 1).

**Table 2:** Concordance between pharmacokinetic and behavioural measures by administration route

ACASI	Oral N=314 (%)		Vaginal N=158 (%)	

Self-reported dosing in past 7 days	No dose	≥1 dose	No dose	≥1 dose

	19 (6%)	295 (94%)	9 (6%)	149 (94%)
PK adherent (within self-reported strata)				
No	**19 (100%)**	197 (67%)	**8 (89%)**	93 (62%)
Yes	0	**98 (33%)**	1 (11%)	**56 (38%)**
Accuracy n/N (%; 95% CI)	118/314 (38%; 33–43%)		64/158 (41%; 33–49%)	

Face-to-Face Interviews (FTFI)	Oral N=306* (%)		Vaginal N=155* (%)	

Self-reported dosing in past 7 days	No dose	**≥**1 dose	No dose	**≥**1 dose

	11 (4%)	295 (96%)	4 (2%)	151 (98%)
PK adherent (within self-reported strata)				
No	**11 (100%)**	197 (67%)	**4 (100%)**	94 (62%)
Yes	0	**98 (33%)**	0	**57 (38%)**
Accuracy n/N (%; 95% CI)	109/306 (36%; 30–41%)		61/155 (39%; 31–47%)	

Pharmacy-returned product counts (PC)	Oral N=305* (%)		Vaginal N = 156* (%)	

Use in the past month by PC	**≤**75%	>75%	**≤**75%	>75%

	25 (8%)	280 (92%)	9 (6%)	147 (94%)
PK adherent (within PC strata)				
No	**21 (84%)**	186 (66%)	**9 (100%)**	89 (61%)
Yes	4 (16%)	**94 (34%)**	0	**58 (39%)**
Accuracy n/N (%; 95% CI)	105/305 (38%; 32–43%)		67/156 (43%; 35–51%)	

We estimated the concordance between estimates of non-adherence by drug PK and self-report (FTFI or ACASI) or pharmacy-returned product counts (PC) in our sample. Accuracy of each mode of self-report was calculated as the percentage of adherence self-reports that agrees with PK test result. Concordance between PK and behavioural data is bolded in the table.

* due to missing data.

### Multivariate logistic regression results

**Figure 4:**
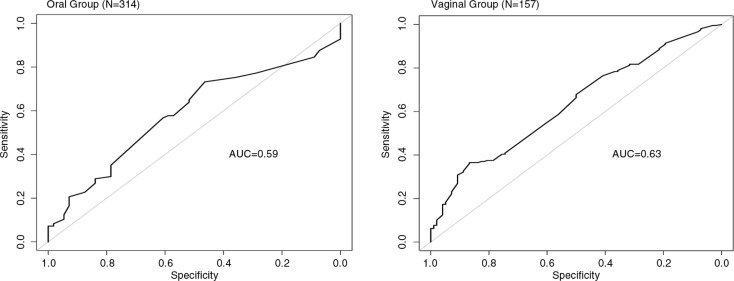
Receiver Operating Characteristic (ROC) Curves for combined behavioural measures of adherence by oral and vaginal groups. Behavioural adherence measures were evaluated using the area under the curve (AUC) to assess the ability of each measure, as well as all measures combined, to correctly discriminate between participants classified as PK adherent versus PK non-adherent. Here, ROC curves are presented based on the sensitivity and specificity of the fitted values from the multivariable logistic regression in predicting adherence with the combined behavioural adherence measures (audio computer-assisted self-interviewing [ACASI], face-to-face interviews [FTFI], pharmacy-returned product counts [PC]). For interpretation, an AUC of 0.50 to 0.60 indicates no discrimination, 0.60 to 0.70 indicates poor discrimination, 0.70 to 0.80 indicates fair discrimination, 0.80 to 0.90 indicates good discrimination and 0.90 to 1.0 indicates excellent discrimination.

We explored the ability of the self-reported measures to predict biologically defined adherence by combining the behavioural measures to predict PK adherence in a logistic regression model. Whether using one behavioural measure or combining all three (FTFI, ACASI and PC) the multivariable models were not much better than chance at predicting PK non-adherence. In the multivariable models, the AUC for participants in the oral group was 0.63 and for those in the gel group it was 0.59 (Figure 4).

### Baseline factors associated with over-reporting product use

The average proportion of obvious over-reporting in the oral group (across month 3-30 visits) was 66% by FTFI and 67% by ACASI, with respect to assessment of product use in the previous seven days (i.e. self-reported using daily with no evidence of product use by TFV concentration). In the vaginal product group, the average proportion of obvious over-reporting (across month 6–15 visits) was 69% by FTFI and 54% by ACASI.

We examined baseline risk factors for over-reporting product use, controlling for time in study and country (Table 3). In the oral group, being married (RR 0.68; 95% CI 0.49 to 0.94) decreased risk of over-reporting, while stating one was “very worried” about getting HIV in the next year (compared to not-at-all worried) was associated with an elevated risk of over-reporting (RR 1.30; 95% CI 1.06 to 1.6). There was a 1% increased risk of over-reporting for each additional month in the study, but time did not remain significant in the multivariable model. Conversely in the vaginal group time was not associated with over-reporting, and indication of being “somewhat worried” about getting HIV (compared with not-at-all worried) was associated with a lower risk of over-reporting (RR 0.42, 95% CI 0.23 to 0.77).

**Table 3:** Correlates of over-reporting recent product use per FTFI, and by administration route

	Oral Group *N*=314					Vaginal Group *N*=158		
		
Baseline Variables	Univariable Adjusted*			Multivariable Adjusted*		Univariable Adjusted*		
			
Country (adjusted for time)	RR	95% CI	*p*	RR (95% CI)	*p*	RR	95% CI	*p*
Uganda vs. South Africa	0.74	0.55 to 0.99	0.04	1.02 (0.72 to 1.44)	0.91	0.78	0.38 to 1.62	0.51
Zimbabwe vs. South Africa	0.80	0.64 to 0.99	0.04	1.16 (0.81 to 1.64)	0.42	0.69	0.41 to 1.18	0.18
**Time** (months)	1.01	1.00 to 1.03	0.04	1.01 (1.00 to 1.03)	0.07	1.03	0.96 to 1.1	0.42
Age over 25 vs. 25 or younger	0.88	0.83 to 1.63	0.12			1.20	0.81 to 1.78	0.36
Primary vs. some secondary school or more	1.16	0.75 to 1.79	0.39			1.26	0.63 to 2.66	0.51
Earns own income; Yes vs. no	1.15	0.98 to 1.35	0.08	1.08 (0.92 to 1.26)	0.36	1.06	0.73 to 1.52	0.77
Married vs. not married	0.61	0.45 to 0.85	<0.02	0.68 (0.49 to 0.94)	0.02	1.08	0.59 to 1.98	0.79
Has a primary partner vs. no primary partner	0.86	0.49 to 1.48	0.58			1.03	0.32 to 3.30	0.96
Living with partner vs. not living with partner	0.85	0.70 to 1.04	0.12			1.24	0.78 to 1.97	0.36
Other male sex partners, past 3 months			0.12					0.64
One vs. zero	0.94	0.77 to 1.15				0.78	0.45 to 1.33	
2+ vs. zero	1.45	0.98 to 2.17				1.06	0.34 to 3.33	
Condom use, last vaginal sex^4^								
Yes vs. no	1.11	0.92 to 1.34	0.28			0.96	0.63 to 1.45	0.84
Anal sex, past 3 months								
Yes vs. no	1.01	0.72 to 1.26	0.73			1.19	0.77 to 1.85	0.43
Sex work in past year^4^								
Yes vs. no	1.13	0.83 to 1.52	0.44			1.1	0.59 to 2.04	0.76
Contraception method^3^								
Oral contraceptive vs. not	0.89	0.72 to 1.08	0.24			1.15	0.74 to 1.79	0.52
Baseline diagnosis of a curable STI^1^								
Yes vs. No	0.98	0.81 to 1.19	0.82			1.15	0.74 to 1.81	0.53
HSV-2 seropositivity^2^ Yes vs. no	0.96	0.82 to 1.12	0.63			1.18	0.82 to 1.72	0.37
Worried about getting HIV in next year			<0.01		0.04			0.02
Very worried vs. not-at-all worried	1.38	1.13 to 1.70		1.30 (1.06 to 1.6)		0.73	0.48 to 1.10	
Somewhat worried vs. not-at-all worried	1.18	0.91 to 1.53		1.14 (0.88 to 1.48)		0.42	0.23 to 0.77	
HIV risk score >6 vs. score of 6 or less	0.96	0.82 to 1.14	0.66			0.94	0.62 to 1.42	0.77

We used a Generalized Linear Mixed Model (GLMM) with random intercept and included all available follow-up quarterly visits with PK measures, adjusting for time (in months) between baseline and PK visit and country. Baseline correlates examined include demographic, sexual behaviour and HIV risk variables, and preselected prior to analysis, as per Table 1.

* All univariable and multivariable analyses are presented, controlling for country and time. Marginal Wald Chi-Square Test *p*-value is provided. If more than one risk factor had a *p*<0.1, they were entered into a multivariable model.

1 and 2 as described in Table 1.

3 Collected by face-to-face Interviews (FTFI) on case report forms.

4 Collected by audio computer-assisted self-interviewing (ACASI).

## Discussion

In this random adherence cohort of 472 VOICE participants assigned to active products, PK thresholds based on route of administration indicated similarly low adherence for women randomized to both the oral and vaginal product arms. This finding is similar to MTN-001, an open-label, cross-over trial of the same products but for a 6-week duration, where adherence estimates differed among US versus African participants, but within sub-Saharan Africa, was similar between products [31]. Among those who behaviourally acknowledged non-use concordance with PK data was very high; however, very few (≤10%) acknowledged low adherence. Thus, none of the behavioural measures we assessed, including FTFI, ACASI or pharmacy-returned PC, accurately predicted PK non-adherence, either alone or in combination. Furthermore, accuracy was no better by ACASI than by FTFI compared with PK data. Over-reporting was widespread in both the oral and vaginal groups, and was not associated with distinct participant characteristics. Specifically, over-reporting was not associated with demographics or a risk score previously developed for assessing risk of HIV acquisition, with the exception of being unmarried, for women in the oral group [11,30]. The sampling for this adherence subset analysis was similar to the full VOICE cohort; hence, findings from this study may be generalizable to all VOICE participants.

To improve on the accuracy of adherence assessed by FTFI, other methods have been evaluated. Minimal misreporting seems to have occurred in the Partners PrEP Study of HIV-serodiscordant couples, where biological evidence, electronic monitoring (MEMS) and other behavioural measures all agreed on high adherence [23]. ACASI did not improve the accuracy of self-reported product use compared with FTFI in VOICE, despite previous research indicating that ACASI is liked by participants and may increase reports of sensitive behaviours. However, in many earlier studies assessing ACASI, there was no biomarker to objectively determine the behaviour of interest; when there was, ACASI did not fare much better than FTFI [32–34]. In the iPrEx trial, CASI and FTFI provided similarly inflated estimates of pill adherence, and were poor predictors of drug detection, with important variations by geographical areas [24]. Thus, in VOICE, iPrEX, Fem-PrEP and MTN-001 (although the latter two trials did not implement ACASI), findings suggest that over-reporting in the context of low product use was common, at least in non-US settings [24,31,35,36]. Self-reports also appeared greatly overestimated when compared to objective assessments of use in earlier microbicide trials, such as the Carraguard trial [37] or in CAPRISA 004 [38,39]. Despite providing greater privacy than FTFI, ACASI may not lead to greater honesty or disclosure of highly socially desirable behaviours, such as product adherence in HIV PrEP trials, in settings where social stigma for HIV and those taking ARV are high, and ambivalence about research abound [24,34,40,41].

Indeed, findings from two qualitative ancillary studies indicate that participants were motivated to enrol and stay in VOICE for the quality health care, regular HIV testing, and other benefits, but were fearful of using the investigational products, which contained ARVs, so that they likely concealed their poor adherence to remain in the trial [40,42,43]. This occurred despite implementing a participant-centred, need-based, non-judgmental approach to product adherence counselling during VOICE, where we were striving for accuracy and honesty [44]. Similarly, in Fem-PrEP, women feared being terminated from the study if they reported their actual behaviour [36]. Given limited or no alternatives for accessing quality health services outside the trial setting, participants may be encouraged to over-report if there is mistrust about the research, no objective means of ascertaining product use and if they perceive negative consequences to telling the truth [24,36,40,42,45].

For our analysis of factors associated with “obvious” over-reporting, we purposefully selected a crude definition (reporting daily use in past week despite no evidence of product use biologically) to minimize misclassification because of recall bias, given that plasma and VF PK data provide average estimates of recent use. Importantly, this low threshold of PK adherence (use within the last week) will not provide any expectation of protection and was selected solely to compare methods of adherence and assess their lack of concordance. Biomarkers that may provide more information on average use over longer term periods, or on white-coat compliance to explain recent use, were not available for our analyses [46,47]. Our definition of over-reporting is different from that used in Fem-PrEP in that we used plasma and VF TFV concentrations and Fem-PrEP used intracellular TFV-DP concentration from the upper layer of packed blood cells with resultant differences in threshold values used to identify poor adherence; this may explain differences between the two studies regarding characteristics of participants who over-reported [48]. In VOICE, over-reporting was not associated with higher HIV risk [11,30] (except for being unmarried in the oral group) or with factors assessing topics known to be subject to social desirability (e.g. condom use, anal sex, number of partners). Neither country nor oral contraceptive use, which was a significant risk factor in Fem-PrEP, was significant here. There also was little evidence for a time effect on over-reporting in VOICE. This may be explained by the sparse biological data, which prevented adjustment by sites (who were activated at different times). Alternatively, PK data collection started relatively late (month 3 in the oral group and month 6 in the vaginal group), presumably when behavioural patterns were already established, both in terms of low product use and over-reporting.

In VOICE, “being worried about getting HIV in next year” was the only factor associated with over-reporting both in the oral and vaginal groups; although, the associations differed in each group. However, this variable may not have accurately measured participants’ risk perceptions. True risk perception may only be one of many factor associated with product adherence and with reporting. Other trials have used different measures of risk perception and the lack of standardization hinders comparisons across prevention trials [49,50]. In iPrEx, men who engaged in higher risk behaviour were more likely to be adherent; however, risk perception was not significantly associated with adherence longitudinally [50]. In Fem-PrEP feeling at risk of getting HIV in the next 4 weeks was not associated with adherence; however, it was marginally associated with over-reporting pill use [48,49]. Qualitative data suggested that HIV risk perception was an important motivator for joining VOICE to access regular testing and health checks, but not necessarily to use the products, hence those motivated to stay in the study may have had many reasons to simply appear adherent [42]. For the vaginal gel group, those who reported being “somewhat” worried about HIV were less likely to over-report. Of note, participants who reported they were “somewhat” worried about HIV were more likely to have plasma PK detected at month 3 in both the oral and vaginal groups [51]. Improvements are needed in validating and standardizing how risk perceptions are measured in PrEP trials. Better measures of risk perception may help elucidate the association, if any, between risk perception, adherence and reporting.

With more accurate electronic or biological measures of product use increasingly available, real-time feedback on adherence can be provided to trial participants [23,52,53]. However, the efficacy of rapid individual feedback with objective data needs to be formally evaluated given the logistical requirements of such real-time intervention, particularly with biomarkers (sample handling, shipping, analysis, inventory, reporting, adherence counselling), which can be a substantial burden on a clinical trial infrastructure. Optimally an objective adherence measure should correlate with protection to fully realize the benefits of real-time monitoring. Furthermore, researchers need to strive to better understand the social and cultural contexts that lead to low product use as well as misreporting to address proactively these barriers when designing future trials. This may include changing procedures at the clinic, influencing the clinical trial culture locally to foster greater trust and generating more engagement to optimize product use and honest reporting from participants. Notwithstanding, accurate real-time, low-cost objective and/or biological measures that minimize opportunities for manipulation or respondent bias are urgently needed to facilitate accurate adherence monitoring and feedback for enhanced support during PrEP and microbicide trials.

## Conclusions

In this study, TFV PK measures indicated similarly low adherence among VOICE participants in both the oral and vaginal product groups. Furthermore, no behavioural measure, regardless of data collection mode, accurately predicted non-adherence, defined by PK. Over-reporting of product use was widespread, and no clear characteristics defined participants who grossly over-reported use of tablets or gel. Objective monitoring of product use should begin early in future trials before behavioural patterns related to low product use and over-reporting are established. Most important, accurate real-time measures to monitor product adherence should become a standard of prevention trial implementation and are key to inform the interpretation of trial results.

## Supplementary Material

Divergent adherence estimates with pharmacokinetic and behavioural measures in the MTN-003 (VOICE) study
