# Supplementary material for: Divergent adherence estimates with pharmacokinetic and behavioural measures in the MTN-003 (VOICE) study
Source: J Int AIDS Soc. 2016 Feb 4;19(1):20642. doi: 10.7448/IAS.19.1.20642 (PMC4744323; doi:10.7448/IAS.19.1.20642)
Supplement: Divergent adherence estimates with pharmacokinetic and behavioural measures in the MTN-003 (VOICE) study [file JIAS-19-20642-s001.pdf]

Additional files:

Additional file 1: Distribution of Accuracy Estimates for Self-reports by Interview Mode (ACASI and FTFI) and Product Mode of Administration (Oral Tablets and Vaginal Gel)

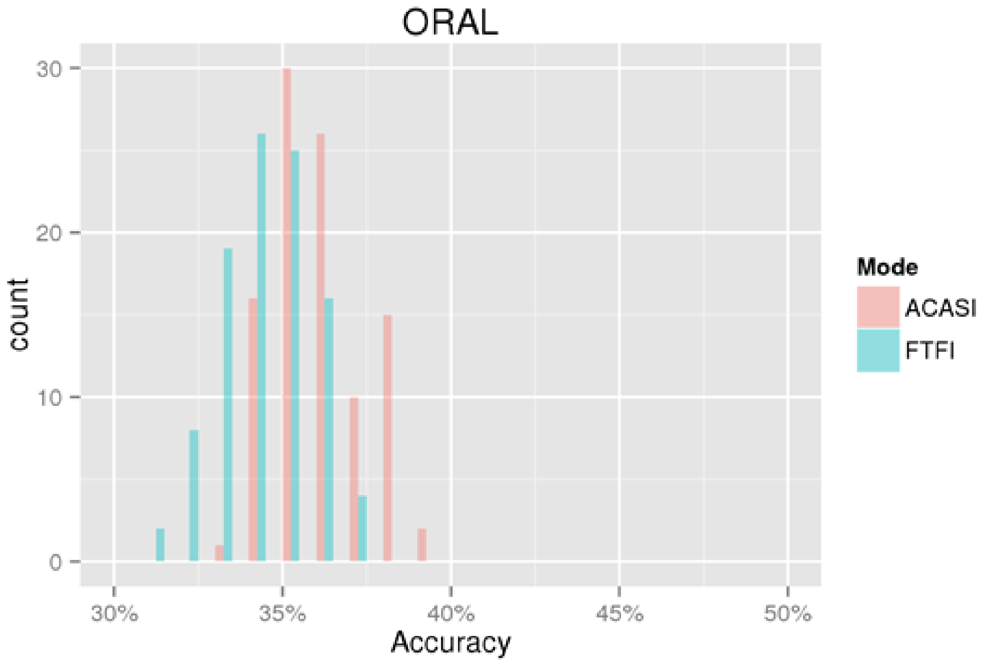

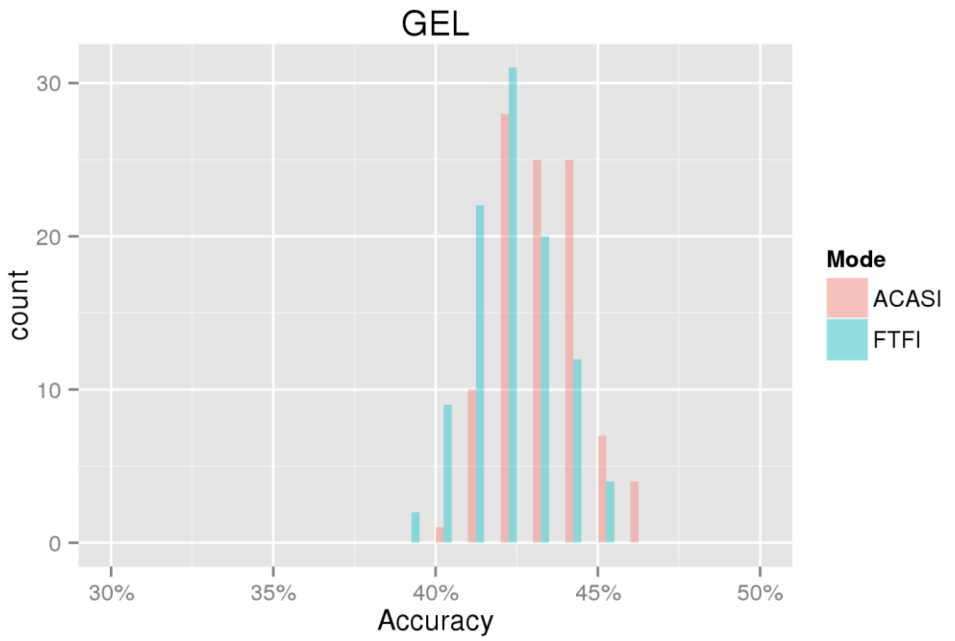

Legend: We conducted a sensitivity analysis of the accuracy of self-reported measures. We randomly selected a visit for each participant, repeated 100 times to create 100 random samples. The chart indicates the distribution of accuracy for each sample. The accuracy reported in the paper falls in the middle of the distribution curve of the random samples for the oral group and towards the lower end of the distribution curve of the random samples for the vaginal gel group.
Note: Y axis is number of samples and X axis is accuracy.
